# Complete genome sequence of *Lactococcus garviae* strain 20-1E isolated from a Danish freshwater moat

**DOI:** 10.1128/mra.00225-26

**Published:** 2026-06-15

**Authors:** Yaovi Mahuton Gildas Hounmanou, Taya Tang, Jørgen J. Leisner

**Affiliations:** 1 Department of Veterinary and Animal Sciences, Faculty of Health and Medical Sciences, University of Copenhagen 555025, Frederiksberg, Denmark; 2 China-Mongolia Biomacromolecule Application "Belt and Road" Joint Laboratory, Inner Mongolia Agricultural University 117454, Hohhot, Inner Mongolia, China; California State University San Marcos, San Marcos, California, USA

**Keywords:** *Lactococcus garvieae*, complete genome sequence, hybrid genome assembly, freshwater isolate, virulence genes

## Abstract

We report the complete genome sequence of *Lactococcus garviae* strain 20-1E, isolated from a Danish freshwater moat. The genome was generated using hybrid Oxford Nanopore and Illumina sequencing. Taxonomic identity was confirmed by GTDB-Tk and average nucleotide identity analysis, highlighting virulence and its potential as a free-living species in freshwater.

## ANNOUNCEMENT

*Lactococcus garviae* is a fish pathogen and may cause human infections (1–3). Strain 20-1E was isolated from a freshwater stream (03 February 2021, Table 1) and sequenced to assess its pathogenic potential. We collected 10 mL water sample with a sterile plastic pipette from approximately 5 cm depth and within 1 m from shore and transferred it into a sterile 13 mL propylene centrifuge tube (TRP, Switzerland) and stored it for less than 48 h below 10°C before arrival at the laboratory.

**TABLE 1:** Sampling location description and genome characteristics for *Lactococcus garviae* 20-1E

Description of location	
Location	E Jutland, Moat, Stagsevold, Stagsrode Forest (4)
Time	3 February 2021
Type	Freshwater stream, pH 6.73, forming a part of a medieval castle moat within a beech forest
Geographic coordinates	55°40′45.7″N 9°50′48.4″E
Genome statistics	
Assembly size (bp)	2,140,176
Number of contigs	1
Contig N50 (bp)	2,140,176
Contig L50	1
GC (%)	38.5%
Number of 5S ribosomal RNA	6
Number of 16S ribosomal RNA	5
Number of 23S ribosomal RNA	5
Number of transfer RNAs	61
Total number of genes	2,111
Total number of CDS^*a*^	2,049
GTDB taxonomy	*Lactococcus garvieae*
Read characteristics	
Illumina reads 1	Total reads: 805,066; total bases: 110,891,409
Illumina reads 2	Total reads: 805,066; total bases: 110,904,335
Genome Coverage Illumina	102×
Nanopore reads	Total reads: 228,521; total bases: 500,778,065; raw reads N50: 7,196
Genome Coverage Nanopore	230×
Read data accession	
BioSample	SAMN54194371
Illumina Reads Accession	SRR36501241
ONT Reads Accession	SRR36502162
Assembly Accession	JBUEDY010000001

^*a*^ CDS, coding sequences.

One milliliter sample was transferred to APT broth (Difco, Sparks, MD, USA), incubated at 15°C under microaerophilic conditions for 3 days, streaked on nitrite-pyridoxal (NP) agar (APT agar [Merck, Darmstadt] with 1 mL 12% sodium nitrite [Ampliqon A/S, Odense, Denmark] and 0.2 mL polymyxin supplement [Oxoid SR 099] per 200 mL agar) and incubated at 15°C for 3 days. Gram-positive, catalase-negative colonies were selected for sequencing.

DNA was extracted using the Bead-Beat Micro AX Gravity kit (cat#106-100-M1; A&A Biotechnology, Gdynia, Poland). Long-read libraries were prepared using the Oxford Nanopore Rapid Barcoding Kit (SQK-RBK114.24) without prior size selection and sequenced on a MinION MK1D device and basecalled using Dorado v0.8.1. Paired-end 151-bp read libraries were sequenced on Illumina NovaSeq X and filtered using Trimmomatic v.0.40 (5), while Nanopore reads were filtered by minimum length of 1,000 bp using Filtlong 0.3.1. Hybrid genome assembly was performed using Trycycler v0.5.6 following the full developer-recommended pipeline (6) (step-by-step guide here: https://github.com/rrwick/Trycycler/wiki/How-to-run-Trycycler), where assembly polishing was conducted using the short reads on the long-read-assembled genomes.

Genome quality and completeness were assessed using CheckM2v1.1.0 and QUASTv5.3.0. Genome coverage was calculated using Qualimapv2.3 based on Minimap2v2.30 read alignments. Taxonomic classification was performed using GTDB-Tkv2.3.2 against the Genome Taxonomy Database (release 214) (7). Genome annotation was carried out using prokaryotic genome annotation pipeline (PGAP v2.0) (8), and ribosomal RNA genes were identified with Barrnapv0.9.

Virulence genes were identified using ABRicatev.1.4.0 with the VFDB v2025 core database, as well as a custom *L. garviae* virulence protein database (https://figshare.com/s/4c5eb3d394b85051380e). Genome assemblies were screened using TBLASTN (BLAST+ v2.17.0), retaining hits with ≥50% amino acid identity, ≥90% query coverage, and an *e*-value 1 × 10⁻²⁰. For each query, the best hit was selected based on bitscore and results summarized as a presence/absence matrix. Antimicrobial resistance and bacteriocin encoding genes were identified using ABRicate with the ResFinder v4.7.2 database and AntiSMASH 6.0 (9) and BAGEL 4 (10).

Average nucleotide identity (ANI) was calculated using FastANI v.1.34 (11) by comparing strain 20-1E against all publicly available *L. garviae* genomes in National Center for Biotechnology Information (NCBI) as of 12 December 2025. Public genomes were retrieved using ncbi-genome-download and dereplicated using dRep v.3.6.2 (11), and a pairwise ANI was visualized as a heatmap in RStudio v4.3.1.

**Fig 1:**
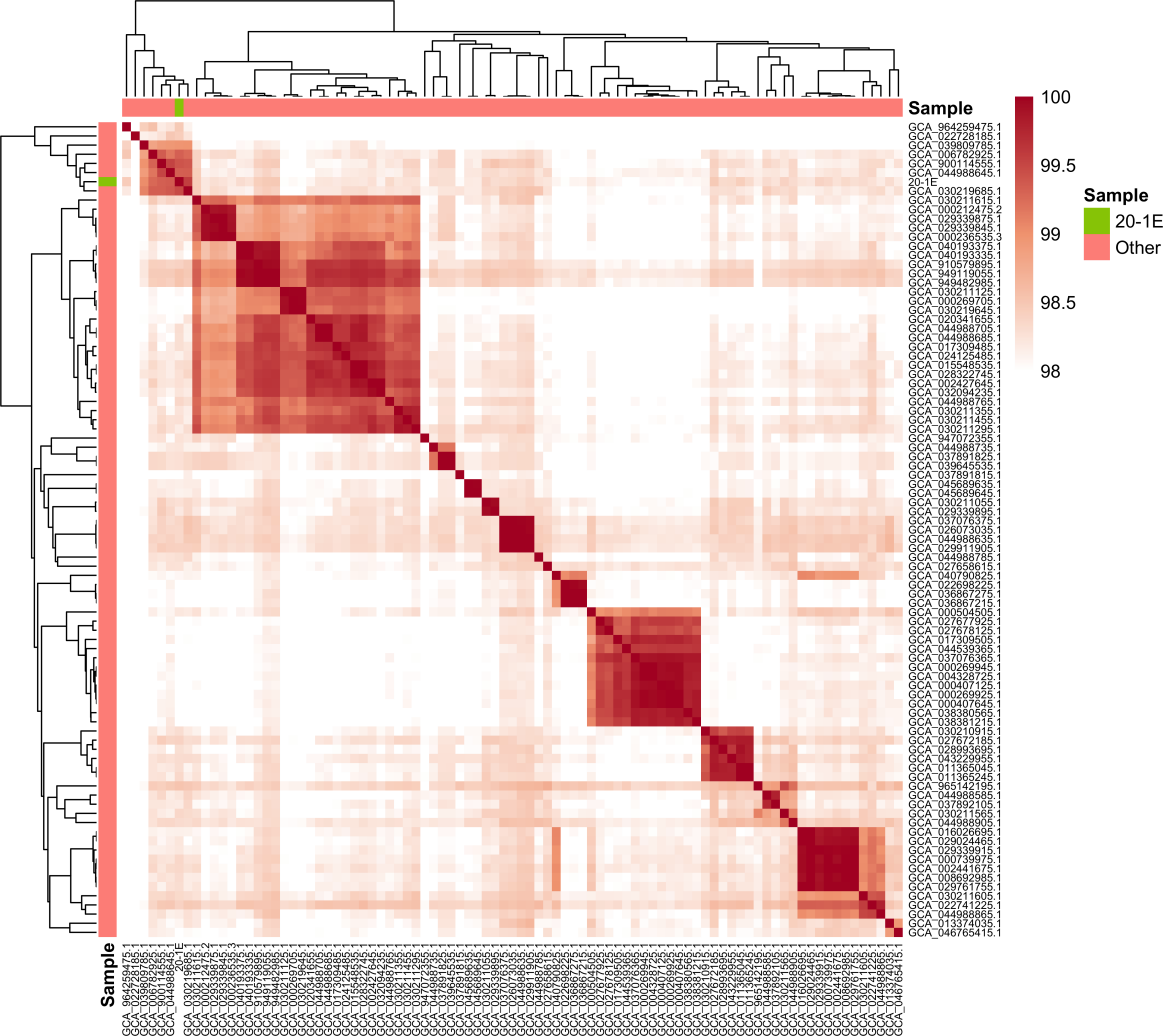
Heatmap of pairwise average nucleotide identity (ANI) of our isolate 20-1E vs public genomes that are within at least 98% ANI to it.

Pairwise ANI analysis (Fig. 1) revealed only four closely related (≥99% ANI) strains: GCA_006782925 (from fish, India, 2019), GCA_030219685 (cattle, Spain, 2023), GCA_044988645.1 (fish, China, 2024), and GCA_900114555.1 (unknown source, 2016).

The complete genome of *L. garviae* strain 20-1E is 2,140,176 bp in length, with a GC content of 38.5%, and encodes 2,103 predicted genes. The genome did not harbor bacteriocin-encoding genes but contained the *mdt(A*) gene, associated with resistance to erythromycin, azithromycin, and tetracycline, and the full constellation of known *L. garviae* virulence genes, including capsule biosynthesis genes, hemolysins (*hly1-hly3*), and multiple LPxTG-anchored adhesins, underscoring pathogenic potential.

## Data Availability

Paired-end Illumina data are submtted to sequence reads archive (SRA) under accession SRR36501241 and Oxford Nanopore Technologies (ONT) sequencing reads have been deposited under SRA accession number SRR36502162. Accession number of the complete genome assembly is available in the Nucleotide’s database in NCBI as JBUEDY000000000 with the full circular chormosome available at https://www.ncbi.nlm.nih.gov/nuccore/CM155296. The ABRicate output for resistance gene and BLAST virulence gene searches are deposited in FigShare with the DOI 10.6084/m9.figshare.31385272.
